# *In-vitro* Study of *Hottentotta Schach* Crude Venom Anticancer Effects on MCF-7 and Vero Cell Lines

**DOI:** 10.22037/ijpr.2020.1100957

**Published:** 2020

**Authors:** Saeed Dezianian, Jamil Zargan, Hamid Reza Goudarzi, Ashkan Haji Noormohamadi, Mohsen Mousavi, Hani Keshavarz Alikhani, Behrooz Johari

**Affiliations:** a *Department of Biology, Faculty of Basic Science, Imam Hossein University, Tehran, Iran. *; b *Human Viral Vaccine Department, Razi Vaccine and Serum Research Institute, Karaj, Iran.*; c *Department of Venomous Animals and Antivenin Production, Razi Vaccine and Serum Research Institute, Karaj, Iran. *; d *Department of Biology, Faculty of Basic Sciences, Razi University, Kermanshah, Iran. *; e *Department of Medical Biotechnology, School of Medicine, Zanjan University of Medical Sciences, Zanjan, Iran.*

**Keywords:** Cancer therapy, Anti-cancer drug, Hottentotta schach, Scorpion crude venom

## Abstract

Scorpion venoms contain potentially useful pharmacological agents. Several studies demonstrate that the venoms of some scorpions induce apoptosis and inhibit the growth of cancer cells; therefore, they have been investigated for isolating anticancer components. In this study, antitumor effects of *Hottentotta schach* crude venom on MCF-7 (breast cancer cell line) as test group and Vero (African green monkey kidney normal cell line) as control group were analyzed. Cell toxicity was analyzed using MTT and neutral red (NR) uptake assays and apoptosis induction was analyzed using comet assay and caspase-3 activity. Oxidative stress following *Hottentotta schach* crude venom treatment was analyzed using nitrite oxide (NO) determination assay, reduced glutathione (GSH) and catalase enzyme activity assays. Results showed that crude venom (25-200 μg/mL) induced apoptosis and inhibited the growth of MCF-7 and to a lesser extent in Vero cell lines. Nitrite oxide concentration increased while glutathione concentration and catalase enzyme activity were decreased in MCF-7 cells; however, results in Vero cells were reversed completely. It can be concluded that *Hottentotta schach* crude venom disturbs the oxidation and reduction potential in cancer cells and ultimately induce apoptosis. So this venom can be used as a good source for isolation and designing new anticancer drugs.

## Introduction

Breast cancer is the most common cancer and the second cancer in the world that causes death. Every year incidence rate of breast cancer increases among women worldwide (1, 2). Although there are many types of cancer treatments such as surgery, chemotherapy and radiation therapy, these methods often prove to be unsuccessful or causing severe side effects (3, 4). In the recent studies introduced differentiation therapy as novel approach that able to irreversibly changing the phenotype of the cancerous cells, can be tried to reactivate the differentiation process and subsequently along with current remedies eliminate the tumor with minimum damage to the normal adjacent cells (5-8). 

On the other hand, studies showed that animal venoms consist of variety of bioactive molecules, small phenolic compounds to high molecular weight polypeptides, and they exhibit almost limitless biological functions. There are about 100 different peptides with different biological properties in the venom of every scorpion species. It is estimated that more than 100000 peptides have been recognized from all scorpion venoms but only 200 peptides were structurally and functionally analyzed (9). *Hottentotta* is the largest genus in Buthidae family and is spread in Africa and Asia continents. More than 90% of dangerous scorpions are members of Buthidae family in the world. *Hottentotta schach* (*H. schach*) is a black, hairy scorpion and 130 mm long and lives in Khuzestan and Fars province in Iran. The venom of *H. schach* contains short chain and low molecular weight neurotoxins (10). Scorpion venom properties depend on genetically variations, geographic areas, and environmental conditions where the scorpions were trapped (11, 12). 

Some peptides in Iranian scorpion venoms have antiproliferative properties and induce cell death and apoptosis in cancer cells. Zargan *et al*. (2011) showed that *Odontobuthus doriae* and *Androctonus crassicauda* crude venom have anticancer properties and induce apoptosis in MCF-7 (breast cancer cell line) and SH-SY5Y (neuroblastoma cell line) cells in vitro condition (13).

In this study, antitumor effects of *H. schach* crude venom and probably mechanism of apoptosis induction in MCF-7 cells as test group and Vero cells as control group were analyzed. 

## Experimental

*Materials*

DMEM-F12 (Gibco, USA), DMEM (Sigma-Aldrich, USA), Trypsin-EDTA (Sigma-Aldrich, USA), Fetal bovine serum (Gibco, USA), EDTA (Sigma-Aldrich, USA), Penicillin–Streptomycin Solution (Sigma-Aldrich, USA), Antibiotic–antimycotic (Invitrogen, USA), PBS (Gibco, USA), Phenol red (Sigma-Aldrich, USA), Trypan blue (Sigma-Aldrich, USA), MTT powder (SRL, India), DMSO [Dimethyl sulfoxide] (Sigma-Aldrich, USA), Triton X-100 (Sigma-Aldrich, USA), Agarose (Sigma-Aldrich, USA), Ethidium bromide (Merc, Germany), Griess reagent (Sigma-Aldrich, USA), Flat Bottom 96-well plate (Sigma-Aldrich, USA), Flat Bottom 24-well plate (Sigma-Aldrich, USA).

*Scorpion venom preparation*

Scorpions were collected from Kazeron in Fars province. Venom was collected by electric shock in telson region of scorpion tails. Venom then was frozen in -50 °C and lyophilized. The lyophilized powder was stored in -20 °C for future use. The powder then was solubilized in DMEM medium (without phenol red) and protein concentration was measured using Bradford method (14).

*Cell culture*

MCF-7 and Vero cell lines were purchased from Iranian biological resource center. The cells were cultured in 75 mL flasks contained DMEM and 10% (v/v) FBS and 10 μL/mL penicillin-streptomycin antibiotics. The cells were incubated in 37 °C, 5% (v/v) CO_2_ and 80% (v/v) humidity (optimum conditions) and media were discarded three times a week. After appropriate confluence, the cells were trypsinized by Trypsin-EDTA and were counted by hemocytometer.

*Cell treatment*

The cells were seeded in 96 and 24-well plate and were incubated overnight in the CO_2_ incubator. After a night of incubation, the media was discarded and the fresh media containing the different concentration (25-200 μg/mL) of crude venom was added to every well. For each test cells were seeded in 96 or 24-well plate and incubated overnight in 5% (v/v) CO_2_, 80% (v/v) humidity, and 37 °C. Then the old media was discarded and new media containing 25, 50, 100, and 200 μg/mL of venom were added and incubated for 24 h in 37 °C. Control group was not treated with venom.

*MTT reduction assay*

The viability effect of scorpion venom was measured by the colorimetric MTT reduction assay (15). 2 × 10^4^ cells were counted by hemocytometer and seeded in 96-well plate and incubated under aforementioned conditions. Then MTT dye with final concentration of 5 mg/mL was added and incubated for 1hr in dark condition at 37 °C. Then, 200 μL DMSO was added to every well and incubated 2 h in dark condition. The absorbance was measured at 595 nm.

*Neutral red (NR) uptake assay*

NR uptake assay was used to estimate the number of viable cells and to confirm the MTT assay results (16). 2 × 10^4^ cells were seeded in 96-well plate and incubated under aforementioned conditions. Amount of 20 μL neutral red dye (5 μg/mL) was added to every well and incubated for 1 h in 37 °C. After red crystals formation, the supernatant was discarded and washed with PBS two times. One-hundred microliter fixation buffer (formaldehyde 37% (v/v), CaCl_2_ 10% (v/v), water) was added to every well and incubated for 1 min and then one-hundred microliter solubilizing buffer (acetic acid 5%) was added and incubated for 20 min in dark condition in a shaker incubator. The absorbance was measured at 540 nm.

*Cells morphology evaluation*

2 × 10^4^ cells were seeded in 96-well plate containing one-hundred microliter media (without serum) and incubated overnight in optimum conditions. Then old media was discarded and new media containing 100 and 200 μg/mL of venom were added and incubated for 24 h at 37 °C. Cells morphology was analyzed and some pictures were captured using inverted microscope (Nikon, Japan).

*Nitric oxide (NO) assay*

Nitric oxide was determined by measuring the nitrite content in culture medium (17). 2 × 10^4^ cells were seeded in 96-well plate and incubated under aforementioned conditions. Then media was transferred to fresh tube and centrifuged in 500 rpm for 5 min in 4 °C. One-hundred microliter media was transferred to fresh 96-well plate and mixed with equal volume of Griess reagent (0.04 g/mL PBS, pH 7.4) and incubated for 10 min at room temperature. Absorbance was measured in 540 nm by a micro plate reader (Biorad, USA). Nitrite oxide concentration (μM/mL) in treated and untreated cells was calculated using nitrite oxide standard curve.

*Reduced glutathione (GSH) assay*

Total reduced glutathione (GSH) was measured following the method of Sedlak and Linsay (18). 5 × 10^5^ cells were seeded in 24-well plate and incubated under aforementioned conditions. The cells then were trypsinized and harvested in fresh 1.5 mL tubes and centrifuged in 1500 rpm for 5 min in 4 °C and washed with PBS (pH 7.4) two times and incubated in -20 °C for 30 min. Two-hundred microliter chilled lysis buffer (NaCl 2.5M, EDTA 100 mM, Tris 10 mM, NaOH 0.2M, Triton X-100 1% and pH 10) was added and incubated for 30 min at room temperature. After 10-15 min sonication, the cells suspension was centrifuged in 2000 rpm for 10 min and a supernatant was transferred to the fresh tubes. The protein concentration was measured using Bradford method and an equal volume of 10% (v/v) TCA was added and stored at 4 °C for 2 h and then centrifuged and a supernatant was transferred to the fresh tubes. Twenty microliter of a supernatant was mixed with 75 μL of lysis buffer, 55 μL of Tris buffer (pH 8.5) containing 0.02M EDTA and 25 μL DTNB (5, 5/dithiobis (2-N benzoic acid)). The absorbance of yellow chromogen was measured using microplate reader. The result was expressed as μg GSH/mg protein using molar extinction coefficient of 13600.

*Catalase enzyme activity assay*

Catalase enzyme activity was measured using cells extract (19). The number of 5 × 10^5^ cells were seeded in 24-well plate and incubated under aforementioned conditions. Then, the cells were trypsinized and harvested in fresh 1.5 mL tubes and centrifuged in 1500 rpm for 5 min in 4 °C and washed with PBS (pH 7.4) two times and incubated in -20 °C for 30 min. Two-hundred microliter chilled lysis buffer (NaCl 2.5M, EDTA 100 mM, Tris 10 mM, NaOH 0.2 M, Triton X-100 1% and pH 10) was added and incubated for 30 min at room temperature. After 10-15 min sonication, the cell suspension was centrifuged in 2000 rpm for 10 min and a supernatant was transferred to the fresh tubes. The protein concentration was measured using Bradford method and 5 μL of the samples was mixed with 50 μL lysis buffer, 20 μL DDW, and 25 μL H_2_O_2_ (15%). The samples were incubated at 37 °C for 2 min and were mixed with one-hundred microliter dichromate acid reagent (0.1 M potassium dichromate in glacial acetic acid) then incubated in boiling water 10-15 min until greenish or faint greenish color was seen. Two-hundred microliter of the samples were transferred to flat 96-well plate and the absorbance was measured in 570 nm using the plate reader. The results were converted into the activity using molar extinction of Catalases (43.6) and expressed as micromoles of hydrogen peroxide consumed/min/mg protein.

*Alkaline comet assay*

Alkaline comet or single-cell gel electrophoresis assay is a good method for DNA fragmentation analysis in the cells (20). 12 × 10^4^ cells were seeded in 24-well plate containing five-hundred microliter media (without serum) and incubated under aforementioned conditions. Then old media was discarded and five-hundred microliter new media containing 25, 50, 100, and 200 μg/mL of venom were added and incubated for 24 h in 37 °C. The cells then trypsinized and harvested in fresh 1.5 mL tubes and centrifuged in 1500 rpm for 5 min in 4 °C and washed with PBS (pH 7.4) two times. Two-hundred microliter PBS was added to tubes and the cells were singled by a needle. The slides were covered by normal melting 1% (v/v) agarose and incubated for 10 min at 4 °C. The cell suspensions were mixed with low melting 1% (v/v) agarose (1 to 2 ratios) and were added to the slides. To form one cell layer, a glass lamel was applied to every slide. In order to cell lysis and nucleus distraction, all slides were incubated for 16-18 h in fresh and cold lysis buffer (NaCl 2.5M, EDTA 100 mM, Tris 10 mM, NaOH 0.2M, Triton X-100 1% and pH 10) at 4 °C. Then, the slides were washed two times with electrophoresis buffer for 20 min and incubated at fresh electrophoresis buffer for 40 min at 4 °C. The electrophoresis was done in 25 V and 300 mA for 45 min at 4 °C. In order to neutralization, the slides were incubated for 10 min in neutralizing buffer (Tris 0.04 M, pH 7.5). Then, the slides were incubated in one-hundred microliter ethidium bromide (20 μg/mL) for 10 min at room temperature. The slides were washed two times (10 min each) with water and analyzed by fluorescent inverted microscope (Nikon, Japan) and the results were statistically analyzed. 

*Caspase-3/CPP32 activity assay*

To determine the caspase 3 activity in MCF-7 and Vero cells treated with *Hottentotta schach* crude venom compared with the untreated control cells, Caspase-3/CPP32 Colorimetric Assay Kit (Catalog #K106-100) was used. The assay is based on spectrophotometric detection of the chromophore p-nitroaniline (pNA) after cleavage from the labeled substrate DEVD-pNA. The pNA light emission was quantified using a microtiter plate reader at 400 nm. Comparison of the absorbance of pNA from an apoptotic sample with an uninduced control allows determination of the fold increase in CPP32 activity.

*Statistical analysis*

The results were reported as mean ± SD and the data were analyzed using GraphPad Prism software. The treated cells and controls were analyzed using ANOVA and *Tukey* tests. The differences were considered to be significant at *P < *0.05 (^*^), *P < *0.01 (^**^), *P < *0.001 (^***^) and *P < *0.0001 (^****^). All the experiments were conducted no less than 3 times.

## Results

*MTT reduction assay*

**Figure 1:**
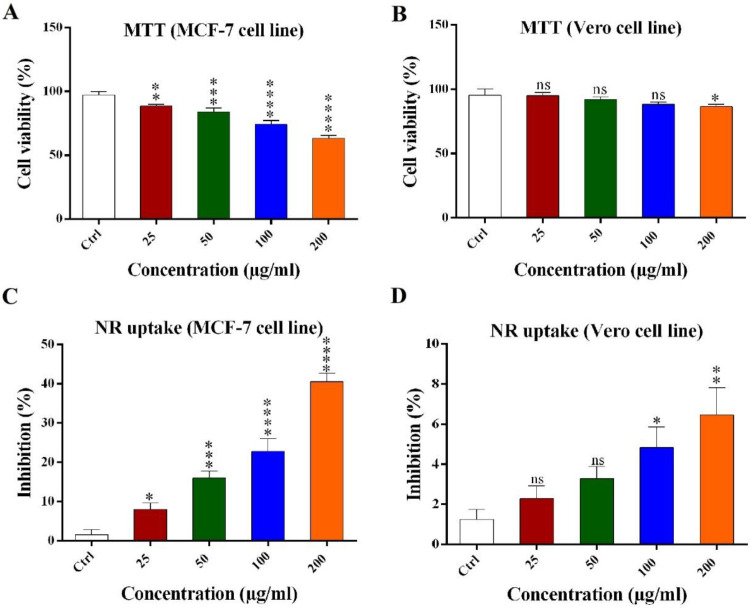
Cytotoxicity effects of crude venom on MCF-7 and Vero cell lines using (A and B) MTT assay and (C and D) neutral red uptake assay. Data were reported as mean ± SD. Results were compared with control. (ns: not significant, ^*^P < 0.05, ^**^P < 0.01, ^***^P < 0.001, ^****^P < 0.0001).

MTT result showed* H. schach* crude venom decreased the viability percent of MCF-7 and to a lesser extent in Vero cells in a dose dependent manner (Figures 1A and 1B). MCF-7 cell line viability was 88.3% (±0.65), 83.8% (±1.2), 77.2% (±1.5), and 72.1% (±0.85) for 25, 50, 100, and 200 μg/mL of crude venom respectively and the Vero cell line viability was 98.8% (±1.6), 94.8% (±1.3), 92.3% (±1.2), and 87.6% (±0.9) for 25, 50, 100, and 200 μg/mL of the crude venom, respectively. The Vero cell line viability percent in 25, 50, 100 μg/mL concentration of the crude venom was not significant but the cell viability was significant for 200 μg/mL concentrations as compared with the control.

*Neutral red (NR) uptake assay*

NR uptake assay was used to confirm the MTT method’s results as showed that the crude venom inhibited the growth of MCF-7 and to a lesser extent in Vero cells in a dose dependent manner (Figures 1C and 1D). MCF-7 cell line inhibition was 9.5% (±2.1), 18% (±1.9), 23.5% (±3.8), and 40.5% (±2.9) for 25, 50, 100, and 200 μg/mL of the crude venom respectively and for Vero cells inhibition was 2.6% (±0.8), 3.4% (±0.76), 4.9% (±0.97), and 6.4% (±1.3) for 25, 50, 100, and 200 μg/mL of the crude venom, respectively. High concentrations of the crude venom (50, 100 and 200 μg/mL) have more inhibitory effects on growth of MCF-7 cell line as compared with the control.

*Cell morphological evaluation*

**Figure 2:**
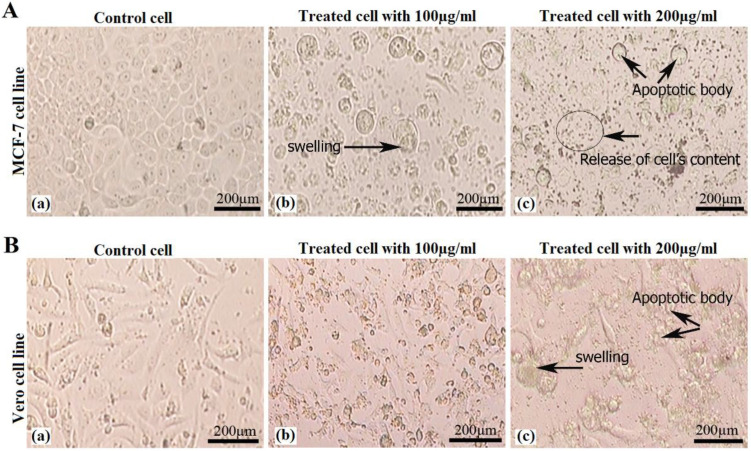
Morphological changes in (A) MCF-7 cell line: control cell, treated cell with 100 µg/mL and treated cell with 200 µg/mL of crude venom, and (B) Vero cell lines: control cell, treated cell with 100 µg/mL and treated cell with 200 µg/mL of crude venom. Pictures of treated cell groups showing increased cell content, swelling and apoptotic bodies (showed with arrows).

The morphology of the treated cells (MCF-7 and Vero cell lines) were changed from spherical shape to multigonal shape and also increased cell content, swelling and apoptotic bodies (Figures 2A and 2B). The morphology of the untreated cells had a spherical shape and no swelling were observed. Results showed that treated cells morphologically differ from untreated cells because of apoptosis happened to the treated cells.

*Nitric oxide (NO) assay*

**Figure 3.**
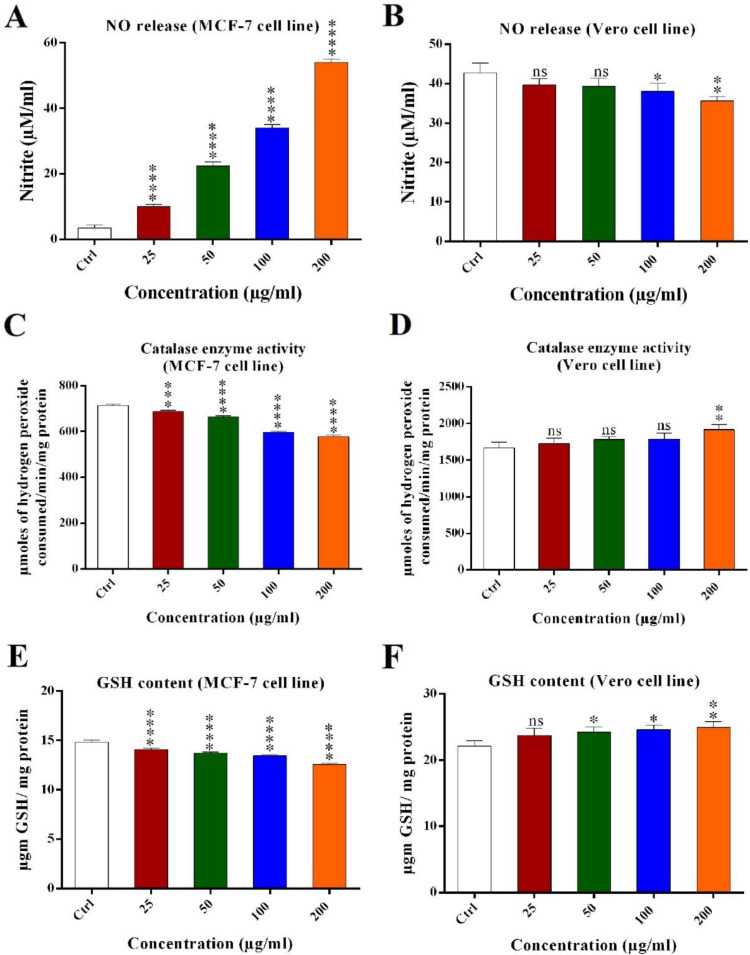
Nitrite oxide released from (A) MCF-7 and (B) Vero cells following crude venom treatment (25, 50, 100 and 200 μg/mL) using NO standard curve. Catalase enzyme activity in (C) MCF-7 and (D) Vero cell line’s extract. Obtained result showed that GSH content of (E) MCF-7 and (F) Vero cell line’s extract treated with 25, 50, 100 and 200 μg/mL of crude venom. The data were reported as mean ± SD and each assay was repeated three times. (ns: not significant, ^*^P < 0.05, ^**^P < 0.01, ^***^P < 0.001, ^****^P < 0.0001).

Scorpion crude venom can increase or decrease the nitrite oxide produced in the cells (21). Nitrite oxide content could be measured from the cell’s media. Scorpion crude venom had increased the nitrite oxide in MCF-7 cells and decreased the nitrite oxide in Vero cells in a dose dependent manner. Nitrite oxide released from MCF-7 and Vero cells treated with different concentrations of crude venom (25, 50, 100 and 200 μg/mL) were 10 (±2.14), 22 (±3.2), 34 (2 ± 8), 54 (±2) μM/mL and 38 (±2.1), 37.3 (±2.7), 35.6 (±2.8), and 34 (±1.4) μM/mL, respectively. Altogether, crude venom significantly induced the release of NO from MCF-7 cells as compared with the control and did not significantly decreased nitrite oxide content of Vero cells in 25 and 50 μg/mL (Figures 3A and 3B).

*Catalase enzyme activity assay*

Catalase enzyme activity was analyzed for MCF-7 and Vero cell lines treated with crude venom. In MCF-7 cells, the activity of catalase enzyme was decreased significantly as scorpion crude venom concentration increased, as compared with control. Catalase enzyme activity were 711.55 (±85), 665.94 (±125), 597.52 (±156), and 577.37 (±138) μmol of hydrogen peroxide consumed/min/mg protein for 25, 50, 100 and 200 μg/mL of crude venom. In Vero cells, the activity of catalase enzyme was not increased significantly in 25, 50, and 100 μg/mL as compared with control and only in the 200 μg/mL of crude venom was significant (Figures 3C and 3D).

*Reduced glutathione (GSH) assay*

GSH content of MCF-7 and Vero cell lines were analyzed after treatment with crude venom. In MCF-7 cells, by increasing the scorpion crude venom concentration, the GSH content of the cells were decreased significantly (*P < *0.001) as compared with control and GSH content were 14.57 (±0.38), 13.7 (±0.22), 13.4 (±0.15), and 12.64 (±0.1) μg GSH/mg protein for 25, 50, 100, and 200 μg/mL of the crude venom. However, the GSH content of Vero cells were increased significantly in all concentrations of crude venom except 25 μg/mL as compared with the control (Figures 3E and 3F).

*Alkaline comet assay*

**Figure 4:**
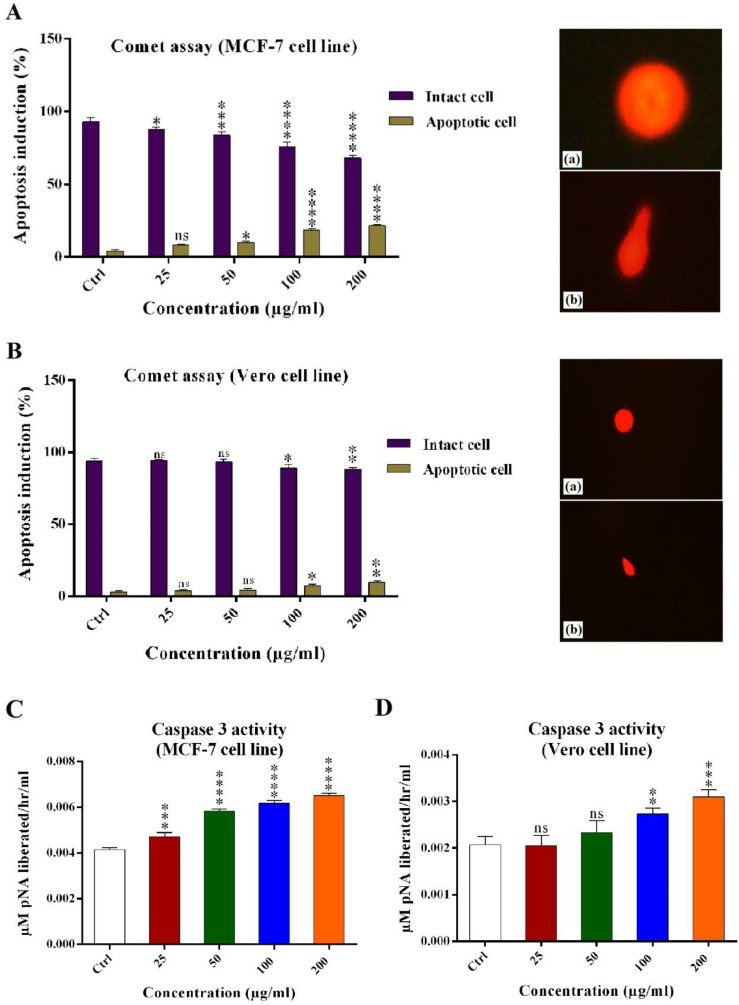
Apoptosis induction analysis effects of crude venom (25, 50, 100 and 200 μg/mL) on (A) MCF-7 cells and its alkaline comet assay pictures: (a) Intact cell and (b) Apoptotic cell. (B) Vero cells and its alkaline comet assay pictures: (a) Intact cell and (b) Apoptotic cell. (C and D) The activity of Caspase-3 enzyme in the MCF-7 and Vero cell lines. Results were compared with negative control. (ns: not significant, ^*^P < 0.05, ^**^P < 0.01, ^***^P < 0.001, ^****^P < 0.0001).

Alkaline comet assay was used for analyzing potential apoptosis induction effects of the crude venom. In MCF-7 cells, apoptosis induction was 7.4% (±0.9), 10.3% (±1.9), 18.9% (±1.8), and 21.8% (±0.87) for 25, 50, 100, and 200 μg/mL of the crude venom, respectively. As a result, the crude venom significantly induced apoptosis in MCF-7 cells as compared with the control (Figure 4A).

In the Vero cells, apoptosis induction was 3% (±0.8), 5.5% (±1.2), 12.6% (±1.1), and 16.4% (±1) for 25, 50, 100, and 200 μg/mL of the crude venom, respectively. In 25, 50 μg/mL of the crude venom, the amount of apoptotic DNA was not significant as compared with the control (Figure 4C).

The DNA images of apoptosis and intact cells were captured using the fluorescent inverted microscope (Nikon, Japan) (Figures 4B and 4D). 

*Caspase-3/CPP32 activity assay*

The activation of Caspase 3/CPP32 was analyzed using Caspase-3/CPP32 Colorimetric Assay Kit which induce apoptosis in the mammalian cells. In MCF-7 cells, Caspase 3 activity was 0.004607, 0.005760, 0.006152, and 0.006447 µM pNa/h/mL for 25, 50, 100, and 200 μg/mL of the crude venom, respectively. Results showed that *H. schach *crude venom significantly increase the activity of Caspase 3/CPP32 which lead to apoptosis induction in MCF-7 cell line. In Vero cells, Caspase 3 activity was 0.002057, 0.002333, 0.002737, and 0.003094 µM pNa/h/mL for 25, 50, 100 and 200 μg/mL of crude venom, respectively. Results showed that *H. schach* crude venom in 25, 50 µg/mL was not significantly increased the activity of Caspase 3/CPP32 in the Vero cell line.

## Discussion

Recent studies showed that scorpion venoms are the source of biologically active molecules which could be a good candidate for antibacterial and anticancer drugs. Some reports showed that scorpion venoms have apoptotic, antiproliferative, cytotoxic, and immunosuppressive properties (21). Apoptosis is a consensus process controlled by genes and is used for eliminating unnecessary and unwanted cells and has a role in immunity and in some diseases (22). The venoms of some scorpions can induce apoptosis and inhibit DNA synthesis in neoplastic cells (17). In this study, apoptosis, antiproliferative, cytotoxic, and oxidative stress effects of *H. schach* crude venom were analyzed on MCF-7 and Vero cell lines. MTT and NR uptake assays showed increased cytotoxicity in MCF-7 and to a lesser extent in Vero cell lines treated with *H. schach* crude venom. These assays were used to indicate cytotoxicity and also used as a marker for the cells membrane intactness (12, 23). Alkaline comet assay showed that *H. schach* crude venom destroys DNA of the cell. The results showed that apoptosis induction in MCF-7 and to a lesser extent in Vero cell lines were increased in a dose dependent manner coordinated with the microscopic analysis. In 2011, Zargan *et al.* showed that *Odontobuthus doriae* crude venom induced apoptosis in MCF-7 cells (24).

In morphological analysis, the apoptotic bodies and swelling were observed in MCF-7 cell line treated with *H. schach* crude venom. For the low concentration of crude venom, the Vero cells were not damaged as compared with MCF-7 cells. However, for 100 and 200 μg/mL of the crude venom, both cells were damaged as seen by inverted microscope. 

Nitrite oxide is a biological molecule which has a role in physiological and pathological processes such as inflammation and cancer. Studies showed that NO in cancer cells induce tumor progression and metastasis (25). Nitrite oxide is produced from L-arginine, NADPH, and oxygen by NO synthase (NOS). NO, like a messenger molecule, regulates the physiological and pathological processes such as cytotoxic functions. Recent studies showed that nitrite oxide can induce tumor progression and metastasis and also inhibit them. The cell’s type and NO concentration in the cells have a role in apoptosis induction or inhibition (26).

iNOS activate apoptosis in the mitoch-ondrial pathway. Our results showed that NO released in the MCF-7 cells treated with *H. schach* crude venom was increased in a dose dependent manner. However, the amount of NO released in Vero cells treated with *H. schach* crude venom decreased. Our results about the MCF-7 cells were coordinated with Zargan *et al.* results (24).

Superoxide dismutase, catalase, and glutathione peroxidase are antioxidant mole-cules in the cells. Catalases convert the H_2_O_2_ to H_2_O and O_2 _(27). This enzyme can be found in liver, kidneys, and red blood cells. Catalase is a tetramer molecule (240 kDa) in mammals which functions in peroxisomes (28). Our results showed that catalase activity in MCF-7 cells treated with *H. schach* crude venom were decreased in a dose dependent manner but increased in Vero cells which showed the cells oxidation and reduction potential were disturbed. 

Reduced glutathione (GSH) is present in all cells and glutathione peroxidase catalyze H_2_O_2_ and oxidized glutathione (GSSG) (29, 30). Our results showed that like catalase, GSH content of the cells were changed. GSH content of MCF-7 cells treated with *H. schach* crude venom were decreased in a dose dependent manner but increased in Vero cells, coordinated with catalase assay results. Zargan *et al.* (2011) results showed that *Odontobuthus doriae* crude venom disrupted the MCF-7 cell’s oxidation and reduction potential (24).

Caspases (cysteine-aspartic proteases) are a family of protease enzymes playing essential roles in programmed cell death including apoptosis (31). In MCF-7 cells, *H. schach* crude venom increased both the activity of Caspase 3 and apoptosis induction in a dose dependent manner. Weiling Li *et al. *showed that the BmK scorpion venom extracts could inhibit the growth of MCF-7 cells by inducing the apoptosis through Caspase 3 up-regulation (32). Also Xiao K.F *et al*. showed that the activity of p21 and Caspase 3 protein expression were upregulated after *Heterometrus liangi* scorpion venom treatment. They also conclude that scorpion venom is to activate the expressions of p21 and caspase-3 protein to cause selective cell apoptosis in Human Kyse-510 Cell (33). Zargan *et al*. also showed that *Androctonus crassicauda* crude venom raise Caspase 3/CPP32 activity in MCF-7 cell line (34).

## Conclusion

It can be concluded that *H. schach* crude venom has cytotoxic effects, inducing apoptosis and inhibiting the cancer cell’s growth. Altogether, *H. schach* crude venom is a good source of molecules for introducing new anticancer drugs. 
